# Bond Shear Tests to Evaluate Different CFRP Shear Strengthening Strategies for I-Shaped Concrete Cross-Sections

**DOI:** 10.3390/ma17133342

**Published:** 2024-07-05

**Authors:** Muhammad Arslan Yaqub, Christoph Czaderski, Stijn Matthys

**Affiliations:** 1 Magnel-Vandepitte Laboratory, Department of Structural Engineering and Building Materials, Ghent University, 9052 Ghent, Belgium; muhammadarslan.yaqub@ugent.be; 2 Empa, Swiss Federal Laboratories for Materials Science and Technology, 8600 Dübendorf, Switzerland; christoph.czaderski@empa.ch

**Keywords:** carbon fibre reinforced polymers (CFRP), I-shaped concrete cross-sections, debonding, anchorages, in-fill blocks, shear strengthening

## Abstract

I-shaped concrete girders are widely used in precast bridge and roof construction, making them a common structural component in existing infrastructure. Despite well-established strengthening techniques using various innovative materials, such as externally bonded carbon fibre reinforced polymer (CFRP) reinforcement, the shear strengthening of an I-shaped concrete girder is not straightforward. Several research studies have shown that externally bonded CFRP reinforcement might exhibit early debonding at the concave corners of the I-shape, resulting in a marginal increase in shear capacity. This research study aims to assess the performance of two different CFRP shear strengthening strategies for I-shaped concrete cross-sections. In the first strategy, CFRP was bonded along the I-shape of the cross-section with the provision of additional anchorage. In the second strategy, the I-shape was transformed into a rectangular shape by using in-fill blocks over which the CFRP was bonded in a U-configuration. In addition to the strengthening strategies, the investigated parameters included two different materials for the in-fill blocks (conventional and aerated concrete) and two different anchoring schemes (bolted steel plate anchor and CFRP spike anchor). To avoid testing on large-scale girders, a new test methodology has been implemented on concrete I-sections. The test results demonstrate the feasibility of comparing different shear strengthening configurations dedicated to I-sections. Among other findings, the results showed that the local transformation of the I-shape to an equivalent rectangular shape could be a viable solution, resulting in shear strength enhancement of 12% to 53% without and with the anchorages, respectively.

## 1. Introduction

I-shaped prestressed concrete girders are commonly used precast structural elements, employed as bridge girders or longer-span (roof) beams in buildings. Strengthening existing structures with such I-shaped girders can become challenging, especially against shear, when an upgrade is necessary due to increased loading, degradation due to impact or ageing, or to fulfil more stringent design provisions. This is because the concave shape of I-sections makes it difficult to reinforce in shear compared to rectangular counterparts. Shear strengthening of I-shaped girders has been researched in the past using externally bonded carbon fibre reinforced polymers (CFRP) in different configurations. However, the problem of early debonding at the web-flange corners has been reported, generally leading to a marginal increase in shear strength unless extra debonding precautions are taken [1]. Indeed, a concave CFRP subjected to tension exerts outbound normal stress on the bond interface, triggering early debonding.

To control or delay FRP debonding in bond-critical applications, several anchorage techniques have been researched in the past [2,3,4,5]. These include transverse FRP sheets or plates at the end of the main FRP reinforcement [6,7], near-surface mounted anchors [7,8], and FRP spike anchors [9]. FRP spike anchors, reported as an effective way of anchoring FRP, have been researched with a focus on pullout behaviour [10,11], bond versus anchored length [12,13], anchor configuration [14,15], as well as design guidance [15,16,17,18,19]. All these anchor types have also been applied to increase the efficiency of shear strengthening systems for I-girders, as briefly detailed in the following.

A comprehensive study [20,21] on scaled I-girders from existing bridges in Canada used vertical strips and reported a 10–18% improvement in shear capacity that became 35% when horizontal strips were employed as anchorage over the vertical strips. Another similar study [22] performed on decommissioned AASHTO bridge I-girders that remained in service for 42 years used vertical CFRP strips anchored using horizontal CFRP strips and reported an increase of 27% in shorter span girders and 15% in longer span girders. A full-scale study [23] reported no shear capacity enhancement due to the complex failure modes of the AASHTO I-girders when strengthened with vertical CFRP strips anchored using horizontal strips or (continuous/discontinuous) CFRP plates bolted on top of the vertical strips at the web-flange corners. Similarly, a comparative study [24] reported no increase in shear capacity of I-girders when vertical CFRP strips were used, anchored with CFRP spike anchors at the web-flange corners. The lack of efficiency was attributed to CFRP debonding between the spike anchors and potential straightening of the CFRP strips, leading to stress concentration at the anchorage regions. When additional horizontal CFRP strips were added to the same strengthening configuration, a shear strength increase of up to 36% was reported. Another study [25] used vertical CFRP strips along with horizontal CFRP strips as anchors at the web-flange corners and reported no increase in shear capacity; however, crack widths were reduced in the strengthened I-girders. For I-girders, a research study [26,27] used vertical CFRP strips without anchorage at two different spacings and reported a 38% increase in shear capacity with strip spacing less than half the effective depth of the beam. In the same study, for wider strip spacing, no increase in shear strength was reported. Another recent study by the authors [28] on large-scale prestressed concrete I-girders used concrete in-fill blocks to transform the I-shape to an equivalent rectangular shape. On top of the in-fill blocks, vertical CFRP sheets in U-configuration were bonded along with CFRP spike anchors in the top flange, reporting an increase in shear capacity of up to 40% with no debonding until failure.

For scaled I-girders of existing bridges in Canada [21], diagonal strips together with horizontal CFRP strips as anchorage reported up to a 36% shear capacity enhancement. Also, a similar strengthening configuration in [22] reported an increase of 22%. Applying a different anchorage configuration, i.e., a CFRP strip epoxied in a groove cut along the web-flange corners, did not improve the shear capacity. Similarly, no shear strength enhancement was reported in [23] using diagonal CFRP strips anchored using bolted CFRP plates at the web-flange corners. Bi-directional continuous CFRP sheets were used in a study [24], anchored using CFRP spike anchors at the web-flange corners, and reported to increase the shear capacity by up to 38%. The shear strength enhancement was not consistent in the case of uniaxial continuous sheets [22] epoxied in a groove cut along the web-flange corners, as one test reported a 36% increase in shear capacity, while the repetition showed no increase due to suspected stress concentration at the web-flange corner from cutting the groove for anchorage. Furthermore, a few supplementary research studies based on numerical modelling [29,30] and analytical analysis [31,32] specifically for the shear strengthening of I-girders using externally bonded FRP reinforcement have also been reported.

Since the available evidence of shear strengthening of I-girders is not consistent but conflicting, and because an alternative strategy could be considered of locally transforming the I-section to a rectangular one, this study aims to assess the performance of different shear strengthening configurations specifically designed for I-girders. Hereby, focus is given to strengthening layouts making use of anchorage systems, rather or not combined with the in-fill blocks. To assess the different strengthening layouts at a medium scale, rather than to be tested on full-scale I-girders, a new test methodology is proposed that is based on the well-established double-lap shear test for bond interaction between CFRP and the concrete substrate.

## 2. Experimental Program

The experimental program employed specially designed specimens, tested in a universal tensile testing machine to assess the relative performance of eight different shear strengthening configurations, in terms of ultimate load, failure mode, and strain behaviour. This exploratory study served as the basis for selecting the most promising shear strengthening configurations to be applied in a follow-up study executed by the authors on full-scale prestressed concrete I-girders [28].

The adopted test methodology is novel in using an I-shaped cross-section with an artificial crack to feature a specific double-lap shear test but basically refers to good practices of using bond shear tests to investigate the FRP interfacial bond behaviour [33] and the relative performance of anchorage systems in externally bonded reinforcement [2,3], as well as earlier work on bond testing in the context of I-girders by [21].

### 2.1. Test Specimen Details

**Figure 1:**
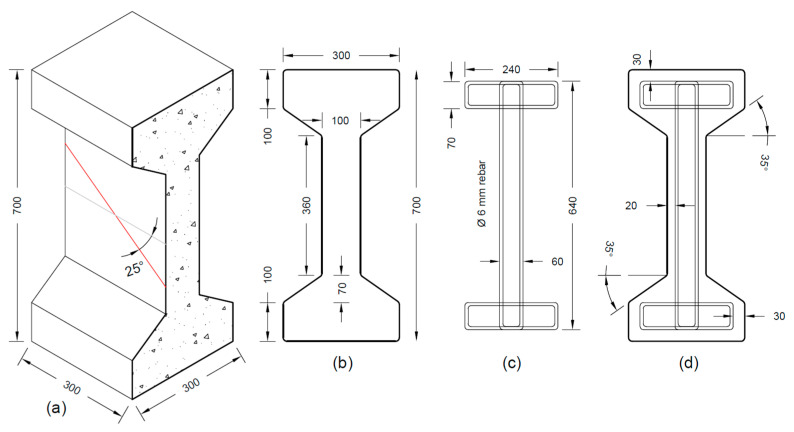
Specimen dimensions (**a**) isometric view; (**b**) side view; (**c**) steel stirrup; (**d**) stirrup inside specimen (dimensions in mm).

The I-section used in this study represents a cross-section that could be exemplary for the precast concrete industry (in Europe, America or other parts of the world), e.g., for constructing concrete bridges and long-span roofs. The detailing of the specimen was mainly governed by considering the dimensions of the tensile testing machine, including the available distance between the clamps gripping the test specimens at both ends, as well as the size of the crosshead. The test specimen has a symmetric geometry as shown in Figure 1a,b, whereby the test specimen represents a slice of 300 mm length that could have been taken from an I-girder, and whereby the specimen can rather or not be reinforced with internal steel stirrups (Figure 1c,d). The symmetrical shape was provided to ensure an equivalent debonding potential on both web-flange corners. The height of the section was 700 mm, while the width and the depth were 300 mm each. To mimic a shear crack passing through the middle of the I-section web, an artificial failure plane was induced in all the specimens using two parallel steel plates cast in the specimen web at an angle of 25° (Figure 1a), which is representative of a typical web shear crack in a prestressed I-girder [34]. These steel plates were welded to Ø20 mm threaded rebars that extended through the end faces of the concrete test specimen so they could be used to grip the specimen in the tensile testing machine (refer to Section 2.5 for further details).

### 2.2. Test Matrix of CFRP Shear Strengthening Configurations

**Figure 2:**
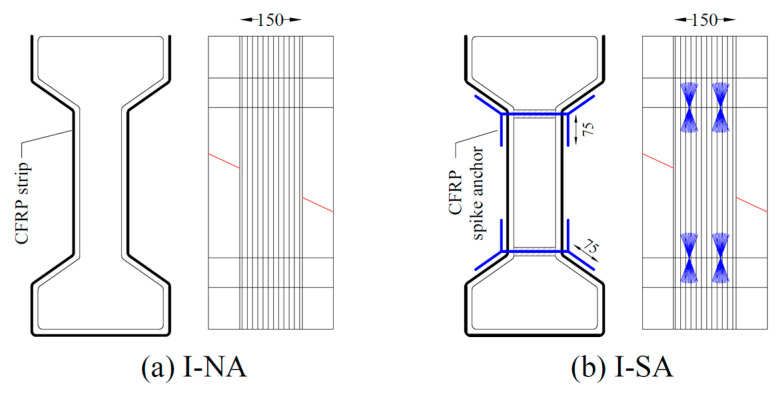
CFRP shear reinforcement along I-shape with (**a**) no anchor and (**b**) spike anchors (dimensions in mm, the anchorages are shown in blue and the predefined failure plane with a red line).

**Figure 3:**
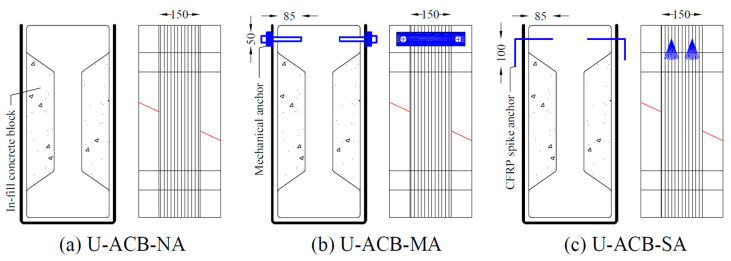
CFRP shear reinforcement along U-shape using aerated concrete in-fill blocks with (**a**) no anchors; (**b**) bolted steel plate anchors; and (**c**) CFRP spike anchors (dimensions in mm, the anchorages are shown in blue and the predefined failure plane with a red line).

The CFRP shear strengthening configurations tested in this experimental program were divided into two basic CFRP strengthening strategies and associated layouts: (i) I-shaped CFRP strengthening (Figure 2, referred to as group G1 in Table 1), being straightforward yet requiring anchorages near the concave shape; and (ii) U-shaped CFRP strengthening through local in-fill blocks (Figure 3), as an alternative to eliminate the debonding due to the concave shape. Local in-fill blocks of aerated concrete (ACB; group G2 in Table 1) or conventional concrete (CB; group G3 in Table 1) have been considered. ACB was considered for its simplicity (lightweight and easy sizing) and to act only as a placeholder for the CFRP. CB was considered for being more compatible with the I-girder (concerning strength and durability) and to act as a sounder surface for the CFRP.

Within each group of specimens, different anchorage configurations are considered. Group G1 consisted of two specimens, where one specimen with no anchor served as a reference (I-NA, Figure 2a), while for the other specimen (I-SA, Figure 2b), spike anchors were provided at the web-flange corners.

Group G2 consisted of three specimens, where aerated concrete blocks (ACB) were bonded to the I-section to transform the I-shape to an equivalent rectangular shape locally in the region where the CFRP strip has to be bonded. The first specimen of this group with no anchors was the reference (U-ACB-NA) as shown in Figure 3a. The second specimen (U-ACB-MA) had bolted steel plate anchors at the ends of the CFRP strip, as shown in Figure 3b. Similarly, for the third specimen (U-ACB-SA), CFRP spike anchors were provided in the top flange to resist debonding as shown in Figure 3c. Note that the spike anchors penetrated the CFRP strip, but the bolts were positioned on both sides of the CFRP strip via a steel spreader plate (dimensions 250 × 50 mm^2^). Group G3 is identical to group G2 (Figure 3), except that instead of aerated concrete blocks (ACB), blocks in conventional concrete (CB) have been used. An overview of all test specimens, their designations, and test parameters is given in Table 1. In the first testing round, 1 layer of CFRP was considered, next to the internal stirrup. In a second testing round, 2 layers of CFRP were considered with no internal stirrup.

### 2.3. Application of CFRP Shear Strengthening Configurations

After casting and subsequent demoulding, the specimens and the in-fill blocks were cured for a month in a wet room (20 °C and >90% RH). The in-fill blocks made of aerated concrete were saw cut from commercially available masonry blocks (size 600 × 250 × 100 mm^3^, by Ytong (Xella BE, Burcht, Beglium)) and bonded to the concrete specimen using proprietary mortar for aerated concrete. Similarly, the in-fill blocks made of concrete were bonded to the specimens using a two-component thixotropic epoxy (Resin 220 HP by S&P Benelux (Tilburg, The Netherlands)). The in-fill blocks (ACB or CB) had a width of 150 mm, equal to that of the CFRP strip. For the first testing round, one CFRP layer (C-sheet 240, 200 g/m^2^ by S&P) was bonded using thixotropic epoxy (Resin 220 HP by S&P) to the concrete substrate and later overcoated with a more fluid epoxy (Resin 55 HP by S&P).

In all the anchored configurations, the anchor holes were drilled prior to bonding the CFRP. In the case of spike anchors at the web-flange corners, CFRP anchors (Ø10 mm) were provided continuous over the web depth, with fans on both sides, as shown in Figure 2b. The fan length equals approximately 75 mm, bonded along both side faces of the concave corner. For the CFRP spike anchors at the top flange, Ø10 mm dowels with an embedment depth of 85 mm were used, with a fan length of 100 mm bonded to the ends of the CFRP strip as shown in Figure 3c. The bolted anchors were made using a steel plate of size 250 × 50 × 5 mm^3^ that was chemically anchored using Ø10 mm bolts embedded to a depth of 85 mm as shown in Figure 3b. The end anchorages were designed following the guidelines available in the literature [17,18,19].

For the second round of tests, the cracked specimens from the first round were reused and strengthened with 2 layers of CFRP. Thus, after execution of testing round one, the tested configurations with one CFRP layer (CFRP strips, anchors, and the in-fill blocks) were removed by grinding and cutting. The two segments of each specimen were properly aligned along the web crack and then held together with temporary steel plates. After bonding the new in-fill blocks and the first CFRP layer (as mentioned earlier), the second CFRP layer was bonded following a wet-on-wet application using the overcoating epoxy. After the application and curing of the strengthening configurations for the second round, the temporary plates were removed when the specimens were clamped inside the tensile testing machine for final testing.

### 2.4. Material Properties

The concrete mixes used for casting the I-sections and the concrete in-fill blocks had a density of 2365 kg/m^3^, Portland cement type CEM I 52.5 N, and gravel with a maximum aggregate size of 16 mm. Table 2 reports the strength of the concrete reference cubes and prisms that were cast, cured, and tested at the same age as the I-sections. The reported compressive strength is the average of three cubes with a side length of 150 mm tested according to NBN EN 12390-3 [35]. The reported flexural tensile strength is the average of three prisms of size 100 × 100 × 400 mm^3^ tested according to NBN EN12390-5 [36]. After performing the flexural tensile tests, the remaining six halves of the prisms were used to perform splitting tensile tests according to NBN EN 12390-6 [37]. The age of the I-sections as well as the reference cubes and prisms at the time of testing was approximately 6 to 7 months. The compressive strength of aerated concrete as defined by the manufacturer is also listed for reference.

The tensile strength of Ø6 mm steel rebar employed for the stirrups, as given in Table 3, was determined by tensile testing and exhibited a yield strength of 570 MPa. The properties of the CFRP strip as defined by the manufacturer are also listed in Table 3, where the nominal (equivalent dry fibre) thickness of one layer of CFRP was 0.113 mm with a tensile strength of 4400 MPa.

### 2.5. Test Setup and Instrumentation

**Figure 4:**
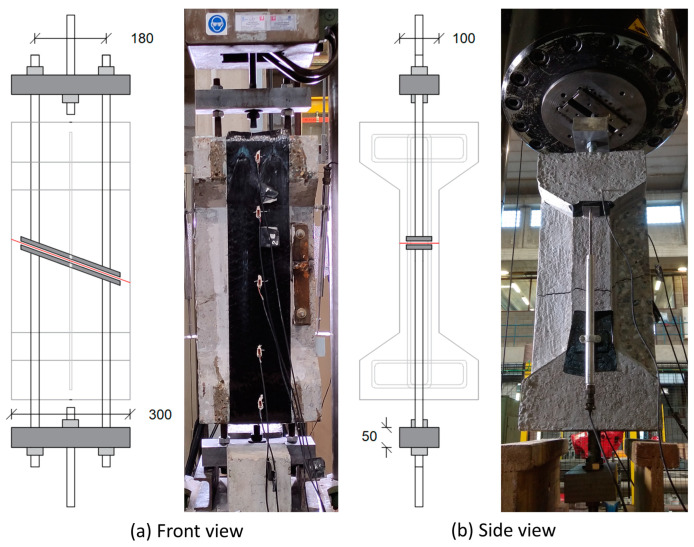
Test setup.

The tests were performed in a universal tensile testing machine with a capacity of 1000 kN, as shown in Figure 4. The threaded bars coming out of the specimens on both ends were fixed to a steel profile of thickness 50 mm and further connected in the middle to a central high-strength threaded rebar (via a spherical seat) that was fixed directly in the clamps of the tensile testing machine. Once the specimen was clamped in the testing machine and instrumented, the load was applied in a displacement-controlled manner at a rate of 0.005 mm/s until failure.

**Figure 5:**
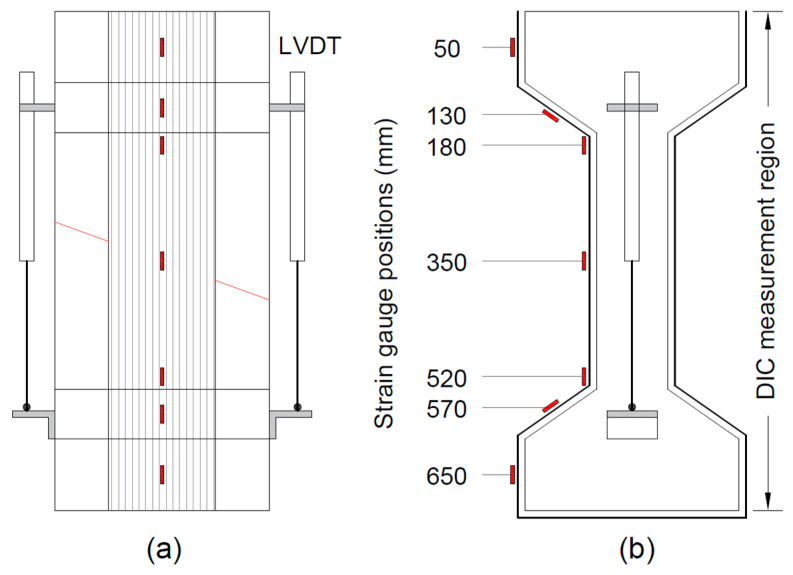
Instrumentation scheme (**a**) front side; (**b**) right side.

The instrumentation scheme used to monitor the response of the specimens is shown in Figure 5. The displacement across the crack was monitored using two linear variable differential transformers (LVDTs) fixed at the sides of the specimens (Figure 5a). To monitor the strain response in I-shaped configurations, strain gauges were installed on one side at seven different locations with respect to the top of the specimens (Figure 5b). For specimens with U-shaped configurations, five strain gauges were used (no strain gauges at 130 mm and 570 mm). On the opposite side of the specimen, non-contact measurements were made using a digital image correlation (DIC) system to observe the deformation field of the applied strengthening system. To this end, a speckle pattern was painted over the specimen side face, which was recorded during testing by means of two cameras.

## 3. Results and Discussion

During a first testing round with the CFRP configurations as presented in Figure 2 and Figure 3, the combination of an internal stirrup and one layer of CFRP was considered, representing a low FRP reinforcement ratio (ρ_f_ = 0.034% and ρ_s_ = 0.063%). The specimens were cracked along the predefined failure plane and the steel stirrups yielded, followed by rupture of the CFRP (and subsequent rupture of the internal stirrup). As for this low ρ_f_ configuration, the failure mode was not debonding but CFRP rupture over the crack, similar failure loads were observed for the non-anchored and the anchored specimens. This did not allow for meaningful observations on the anchorage behaviour, but the results confirmed the feasibility of both strengthening strategies. Specimens I-NA, U-ACB-NA, and U-CB-NA failed at 100 kN, 77 kN, and 108 kN, respectively, indicating similar capacity for both strategies, in reference to CB in-fill blocks as the ACB specimens underperformed. Therefore, in the following, the results of the first round of tests are not presented and focus is given only on the results of the second round of tests, where the cracked specimens from the first round were reused and strengthened with 2 layers of CFRP. These latter specimens (with ρ_f_ = 0.068% and ρ_s_ = 0.00%) failed mainly by debonding and allowed for a better comparison of the different configurations.

### 3.1. Ultimate Loads and Failure Modes

**Figure 6:**
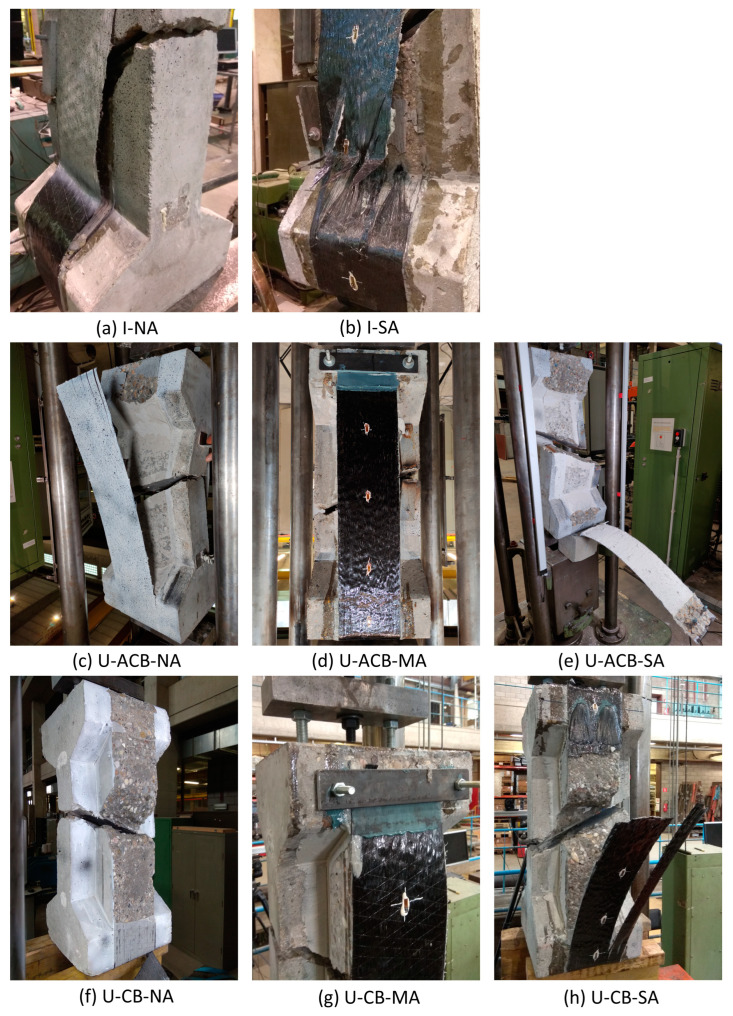
Failure modes of the different specimens.

The main test results in terms of ultimate load, strengthening degree, and failure mode are given in Table 4. The G1 reference specimen I-NA failed by CFRP debonding at the lower web-flange corner (Figure 6a). In the case of a similar anchored configuration I-SA, the spike anchors were able to postpone the debonding at the web-flange corners, resulting in CFRP rupture (as well as rupture of one of the spikes) at the location of the spike anchors near the lower flange (Figure 6b). Thanks to the spike anchors, an increase of 36% in the ultimate load was achieved for I-SA compared to I-NA, and a resulting shift in failure mode from debonding to a mixed mode of CFRP rupture/anchor failure.

Comparing specimens in G1 and G2, the latter configuration showed mostly higher ultimate loads, attributed to the higher efficiency of the U-shape versus the I-shaped CFRP. Specimens U-ACB-NA and U-ACB-MA failed with debonding of the CFRP strips as shown in Figure 6d,e, where in the case of U-ACB-MA, the CFRP strip slipped under the bolted anchor after debonding with a 9% higher load compared to U-ACB-NA. The failure mode of U-ACB-SA was rather unique and involved the rupture of the concrete flange with the pull-out of the spike anchor dowels. Post-testing assessment revealed that the embedment depth of the dowels was less compared to the design details shown in Figure 3c; nevertheless, a 28% increase in the ultimate load was reported compared to the reference U-ACB-NA, demonstrating the effectiveness of the spike anchors.

Among all tested configurations, the best performance with respect to ultimate loads was obtained for G3 specimens with concrete in-fill blocks, for which the failure modes are shown in Figure 6f–h. It can be seen that U-CB-NA failed by the full debonding of the CFRP strip over the concrete in-fill block (Figure 6f) and U-CB-MA also failed with CFRP debonding, yet at a 30% higher load level and, at that point, by slippage under the bolted anchor (Figure 6g). The last specimen, U-CB-SA, failed by rupture of the CFRP just under the spike anchors, where the CFRP strip has a thin cross-section compared to the CFRP in the anchorage region, resulting in strain concentrations (Figure 6h). The increase in the ultimate load of U-CB-SA is 37% compared to U-CB-NA. Indeed, the failure modes of the specimens in G2 and G3 were quite similar, though with about 18% and 7% higher failure loads for G3 specimens, for the bolted anchor and spike anchor configurations, respectively.

### 3.2. Crack Bridging Behaviour

#### 3.2.1. Crack Opening

**Figure 7:**
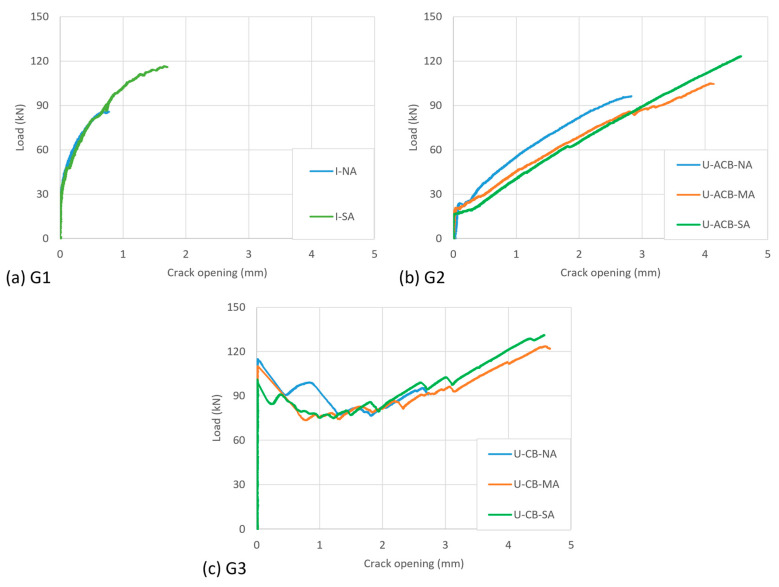
Crack opening versus applied load (for interpretation of colours in the figure legend, the reader is referred to the web version of this article).

The displacement at the middle of the specimen across the crack (average of the two LVDTs) is mainly governed by the crack opening and is plotted against the load for all the specimens in Figure 7. For groups G1 to G3, the crack opening as a function of load appeared very similar per group of specimens, with some difference near the ultimate given the differences in observed failure aspects.

**Figure 8:**
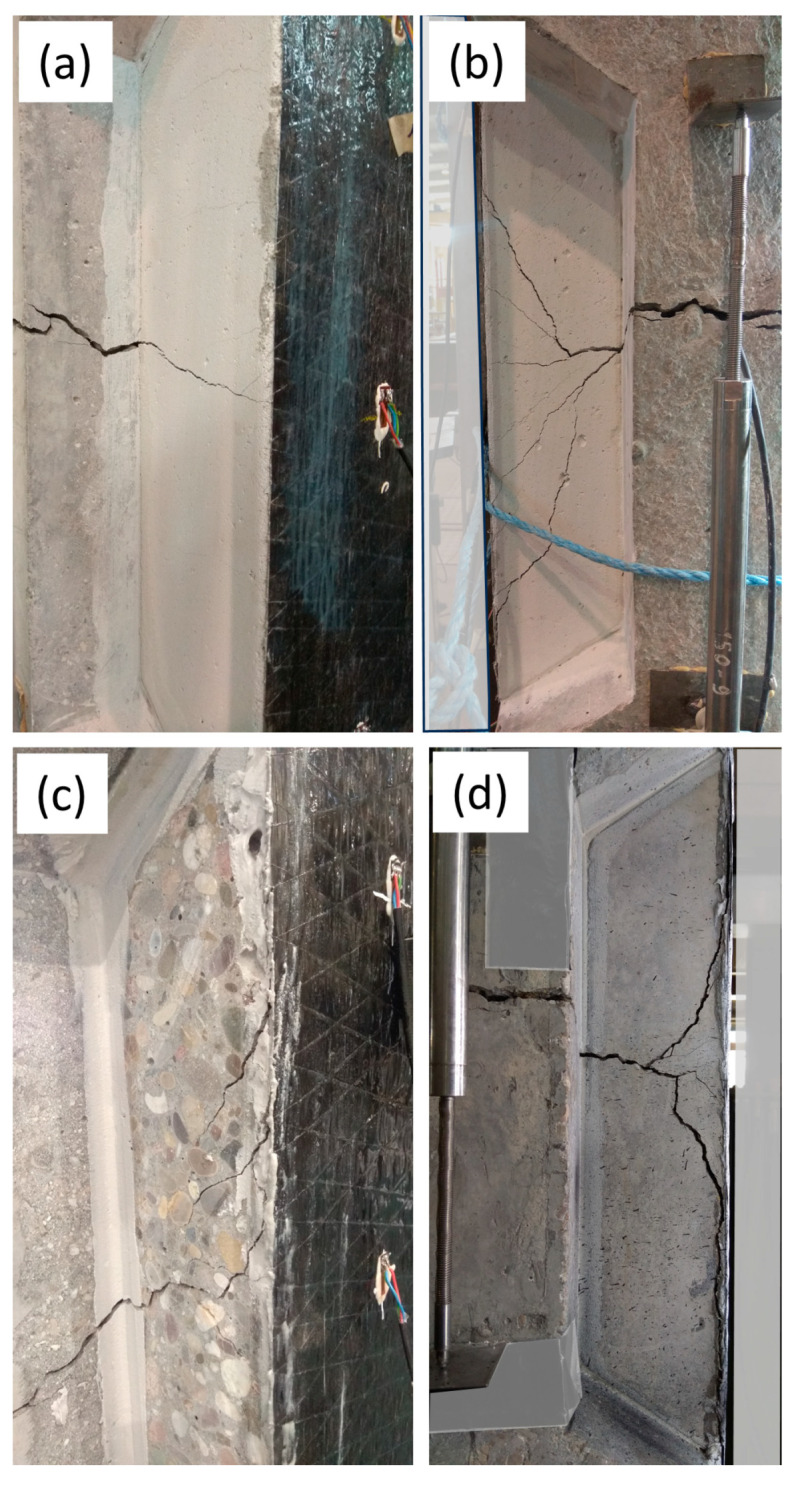
Cracking of the bonded in-fill blocks: (**a**) web crack extending into ACB; (**b**) multiple cracking in ACB; (**c**) web crack extending into CB; (**d**) multiple cracking in CB.

In I-shaped configurations, the load over the specimen was directly taken by the CFRP strips, and the crack started to open around 30 kN. In U-shaped configurations, it was significantly influenced by the cracking of the in-fill blocks. The aerated concrete, being weak in tension, did not significantly delay the crack opening (Figure 7a versus Figure 7b), but in the case of epoxy-bonded concrete in-fill blocks, the crack opening was delayed by a factor of 3 to 4 (Figure 7a versus Figure 7c). The cracking in the bonded in-fill blocks started exactly at the location of the existing crack (Figure 8a,c). Initially, a single crack appeared that later, due to the presence of the CFRP, extended into more distributed cracking (multiple cracks as shown in Figure 8b,d).

The stiffness of G1 specimens (Figure 7a) gradually decreased in terms of crack opening as a function of increasing load. The development of debonding damage resulted in a further reduced slope of the curves near ultimate. Comparing the specimens with I-shaped CFRP (G1, Figure 7a) with those of U-shaped CFRP by means of ACBs (G2, Figure 7b), the latter has a less stiff crack bridging behaviour (than when the CFRP is directly bonded to the web surface), resulting in larger crack openings for a given load. Specimens G3, upon cracking of concrete in-fill blocks, showed a load drop. This is associated with the high cracking load, after which the loads were redistributed to the CFRP (with a corresponding crack opening of about 1 mm). The CFRP resisted higher loads as the crack opening further increased until CFRP debonding/anchorage failure. The slope of the crack opening curves of specimens G3 is at that point similar to that of specimens G2.

#### 3.2.2. Strains at the Level of the Crack

**Figure 9:**
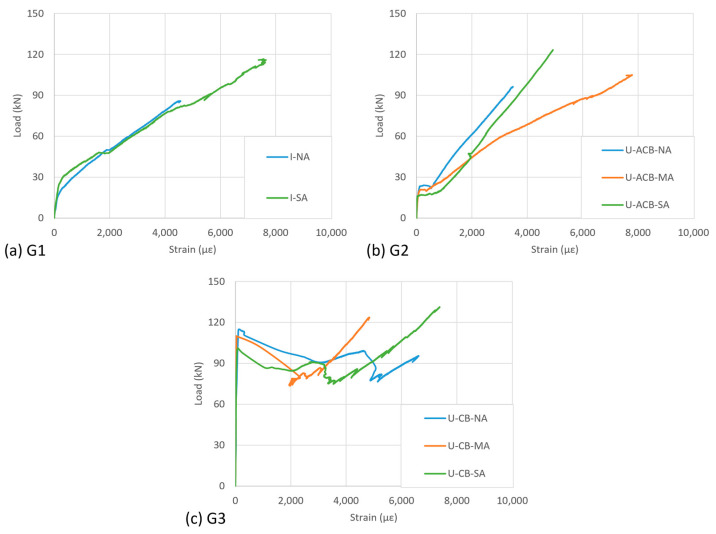
Comparison of strains at the level of the crack (for interpretation of colours in the figure legend, the reader is referred to the web version of this article).

With reference to the observed crack opening behaviour (Figure 7), high strain concentrations can be expected in the CFRP at the crack. This is expressed in Figure 9, at mid-height of the specimen (corresponding to the strain gauge at 350 mm indicated in Figure 5) where the web crack is located. The recorded CFRP strain indeed corresponds to that of the crack opening (Figure 7). The localised strain development at increasing load, of the specimens within each group, is fairly consistent up to a certain load level. Afterwards, deviations in the strain at mid-height become more prominent, associated with reaching ultimate with different behaviour of the strengthening layouts and anchorage systems. Generally, higher CFRP strain levels are developed at mid-height for the anchored specimens. The observed strains at mid-height near ultimate are in the order of magnitude of 3500 με up to 7500 με, which corresponds to 19% (non-anchored specimens) up to 42% (anchored specimens) of the ultimate strain capacity of the CFRP in direct tensile testing and being controlled by failure mechanisms happening at locations other than mid-height.

### 3.3. CFRP Strain Distribution

The CFRP restraining the crack opening in I-shaped (G1) or U-shaped (G2, G3) configurations has a strain distribution along its length that is further influenced by concave or convex corners, local discontinuities introduced by the in-fill blocks, and the presence of extra anchorages. These observations are given in the following as strain profiles along the height of the specimens and the strain field at the peak load.

#### 3.3.1. Strain Distribution along the Height

**Figure 10:**
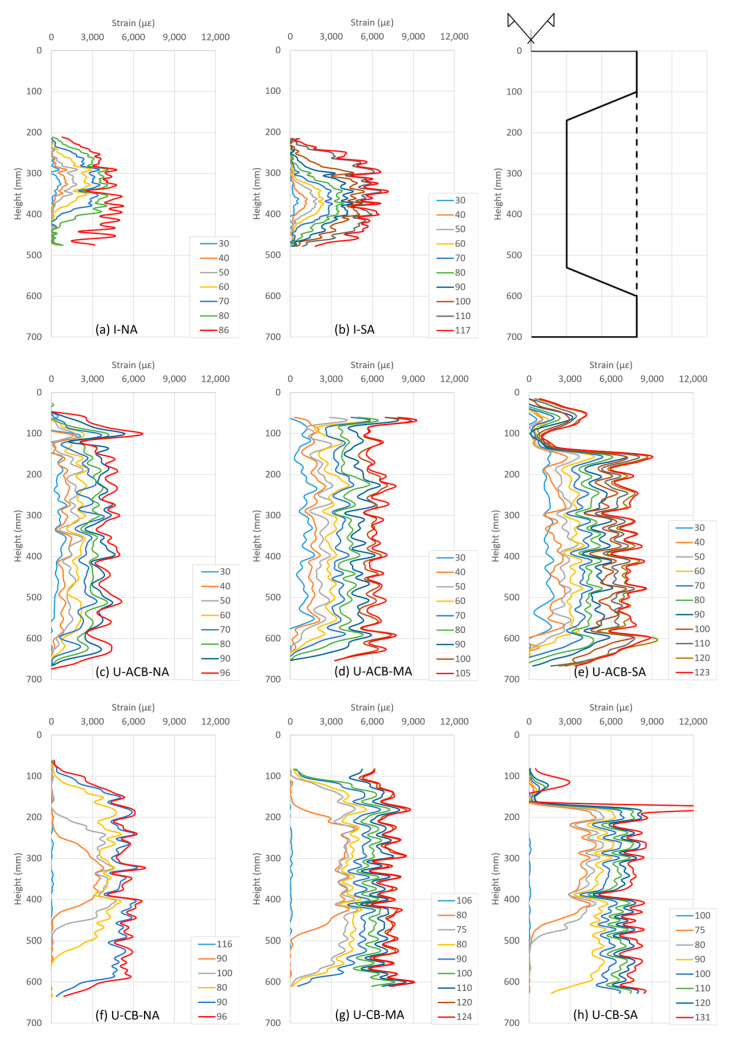
Strain variation in CFRP along the height of the specimens at different load levels in kN.

The FRP strain variation along the height of the specimens (in the centre line of the FRP strip as recorded by DIC) at different load levels is shown in Figure 10. For the I-shaped configurations, it can be seen in Figure 10a,b that the strains are concentrated in the region around the crack. With each incremental load, strain increases in such a way that the peak value remains over the location of the crack yet develops strains over a major part of the web. The strain gauges on FRP regions out of the field of view of the DIC, i.e., the inclined part of the flanges (130 mm, 570 mm) and the side of the flanges (50 mm, 650 mm), did not show any increase in FRP strains up to failure, meaning that CFRP in those regions did not contribute against the applied loads. Indeed, specimen I-NA failed at the lower concave corner as can also be observed from the strain distribution in Figure 10a at 86 kN. For specimen I-SA, the strain distribution near ultimate remains between the fans of the spike anchors, whereas the lower anchorage controlled ultimate.

In specimens with in-fill ACBs, just after the cracking of the ACBs, the strains started to develop over almost the full height as shown in Figure 10c–e. This led to a direct force transfer to the concrete flanges, where debonding failure was susceptible due to the limited FRP bond length (at the top flange). The provided bolted steel plate anchor as well as the spike anchor helped in resisting the debonding, and the resulting strain concentration just under the anchor locations can be seen in Figure 10d,e.

For G3 specimens, it can be seen in Figure 10f–h that just before the cracking of the concrete in-fill blocks, there were no strains in the FRP. Since the load was high at the onset of cracking in the concrete in-fill blocks, the strain in FRP abruptly increased in the region of the crack with a gradual spread over the full length of the concrete in-fill block with increasing load. The strain trend in the vicinity of the anchorages (Figure 10g,h) remains the same as observed earlier for the specimens with in-fill ACBs (Figure 10d,e).

#### 3.3.2. Strain Field at Peak Load

**Figure 11:**
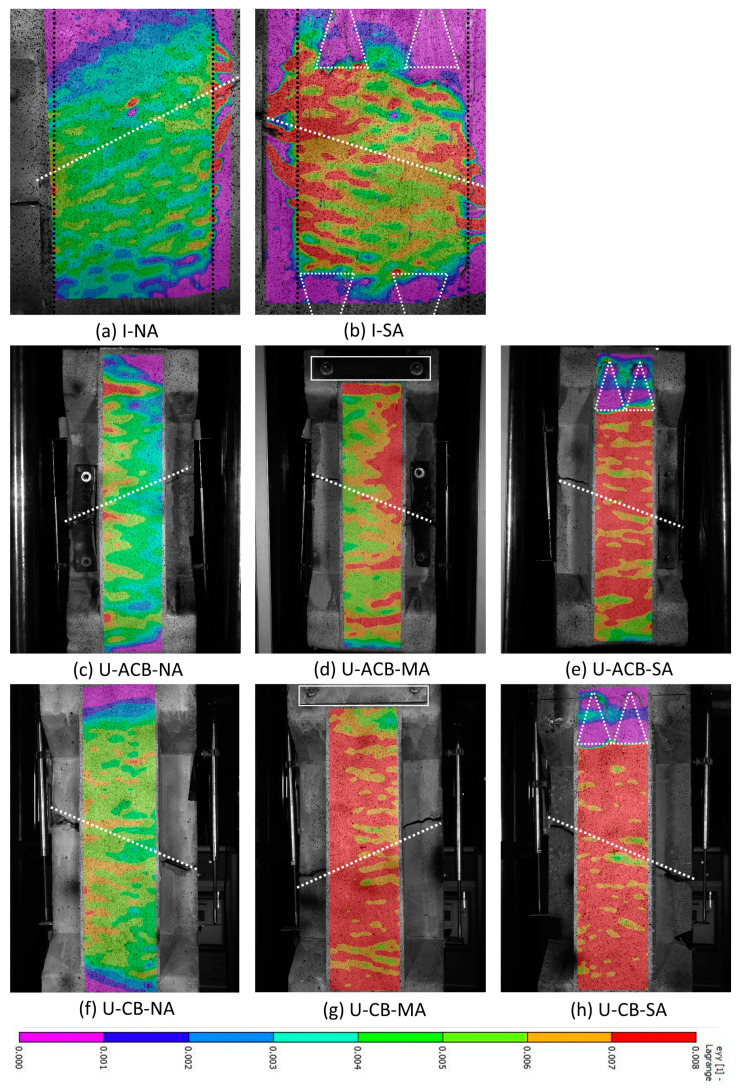
CFRP strain distribution at peak load (white lines represent the location of the anchors and the failure plane).

The strain field at peak load observed using DIC over the full width of the CFRP strips is shown in Figure 11, over an identical contour colour legend. For G1 specimens in Figure 11a,b, the strain concentration can be seen over the crack. For specimen I-NA, Figure 11a shows the strain development towards the lower web-flange corner where the debonding occurred. In comparison, the spike anchors resisted this strain development towards the web-flange corner as seen in Figure 11b for I-SA, resulting in delayed debonding and thus higher strain values. The spike anchors provided extra FRP area, resulting in lower strains in the anchored zones as seen in Figure 11b.

The strain contours for specimens with in-fill ACBs at peak loads are shown in Figure 11c–e, where a more uniform strain distribution can be seen over the full height. For U-ACB-NA, the subsequent strain concentration over the short bond length near the top flange where the debonding occurred can be seen in Figure 11c. The anchorage regions resist strain development towards the ends of the FRP, and therefore, the strain concentration occurs in the FRP just outside the anchorage region as can be seen for the bolted anchor in Figure 11d and for the spike anchors in Figure 11e.

Similarly, the FRP strain distribution for the specimens with concrete in-fill blocks is shown in Figure 11f–h. Compared to the I-shaped configuration and the U-shaped configuration with in-fill ACBs, this configuration helped in developing the highest strains/loads in the FRP, showing a greater concentration of red contours. The behaviour of the anchorage zones for this configuration also seems similar to that demonstrated for the U-shaped configurations with in-fill ACBs.

## 4. Observations and Recommendations

### 4.1. Effect of Investigated Parameters

From the strengthening degrees listed in Table 4 (column 5), it is obvious that the U-shaped configuration (with both types of in-fill blocks) exhibits higher ultimate loads compared to the equivalent I-shaped configuration. This is because the transformation of the I-shape to a standard U-shape using bonded in-fill blocks eliminated the local debonding of the CFRP at the web-flange corners. As seen earlier in the test results, there is a clear difference in the behaviour of the U-shaped configurations with in-fill aerated concrete blocks and those with concrete in-fill blocks. However, when comparing unanchored specimens U-ACB-NA and U-CB-NA, the ultimate load of both types was in the same order of magnitude, demonstrating equivalent potential. Comparing ACB and CB combined with anchorages, the CB specimens performed better as these stronger in-fill blocks positively influenced the anchorage behaviour.

To understand the smaller crack openings in the I-shaped configuration compared to the U-shaped configurations, reference can be made to the strain distributions over the height of the specimens (Figure 10). Considering that the load of the cracked specimen is basically taken by the CFRP, to achieve a certain load level, each configuration requires a similar strain magnitude, as can also be observed in Figure 10. As the crack opening is controlled both by the CFRP strain value as well as the height over which this strain develops, higher crack openings can be expected in the case of more uniformly distributed strain profiles as observed for the configurations with in-fill blocks.

The comparison presented in Table 4 shows that the anchored configurations reach a higher ultimate load than the unanchored configurations. In the case of the bolted anchor, the failure mode was never the rupture of the CFRP strip but CFRP slippage under the steel plate. Such an anchorage configuration appeared, for the configurations in this study, less effective than the spike anchors.

The CFRP spike anchors provided continuity to the CFRP strip, and the failure mode was shifted from end debonding to rupture of the CFRP strip at the spike anchor. The embedded part (dowel) of the spike anchor provided deeper force transfer to the concrete substrate, while the external part (fan) provided the mechanism of force transfer between the spike anchor and the CFRP strip. The increase in density of the fibres in the anchorage region decreases the CFRP strains, which in turn results in strain concentration in the CFRP strips just outside the anchorage region where the rupture occurred. In the current study, the CFRP spike anchors both in the compression flange (with concrete in-fill blocks) as well as at the web-flange corners exhibit a reasonable increase in the ultimate load, i.e., 53% and 36%, respectively. This trend is in line with another study executed by the authors on full-scale I-girders [28], where the concrete in-fill blocks with CFRP spike anchors in the compression flange resulted in a 40% increase in shear strength, whereas the CFRP spike anchors at the web-flange corners resulted in a 12% increase in shear strength. This limited efficiency of the CFRP spike anchors at the web-flange corners of I-shaped strengthening configurations is also demonstrated in another large-scale study on AASHTO I-girders [24]. It might relate to the fact that spike anchors installed in a thin web, on the one hand, increase the bond capacity, but on the other hand, weaken the web. On the other hand, a properly anchored I-shaped shear strengthening configuration tends to be stiffer (less crack opening) compared to its counterpart with in-fill blocks and properly anchored U-shape shear strengthening.

Note that the location of the critical crack considered in this experimental campaign is at the middle of the web, providing maximum FRP bond length on either side of the crack. The efficiency of the I-shaped FRP strengthening configurations may further be reduced if the shear cracks appear closer to the web-flange corners. However, properly anchored U-shaped configurations are analogous to fully wrapped systems, which are more effective in bridging the cracks irrespective of the crack location.

### 4.2. Observations Regarding the Test Methodology

In this work, a methodology has been implemented to evaluate shear strengthening configurations for I-shaped girders by means of a dedicated bond shear test. It is important to mention that these bond tests, performed on a special I-shaped specimen configuration, cannot represent actual shear tests on I-girders. This is because the performance of externally bonded reinforcement in shear tests is also influenced by the interplay of various shear strength contributors, i.e., aggregate interlock, dowel action of longitudinal reinforcement, resistance of the compression zone, and the presence of shear reinforcement. Despite these constraints, the conducted tests were able to capture important behavioural aspects between the studied configurations, enabling decision-making and fine-tuning of full-scale experimental campaigns requiring large resources, such as in the follow-up study by the authors [28].

### 4.3. Tentative Recommendations

The installation of the presented shear strengthening systems in this work is realistic with respect to onsite applications, provided there is space available between the girders for the procedural interventions.

Since externally bonded CFRP shear reinforcement over I-shaped cross-sections is susceptible to early debonding, it is not recommended as a practical solution unless proper anchorage systems are implemented. Adding anchorages at the web-flange corners helps in delaying the debonding but remains challenging, as the increase in efficiency might still be limited depending on the situation (e.g., because not all anchor systems have been proven effective and because drilled anchors can weaken the thin web). On the other hand, the local transformation of the I-shaped cross-section to an equivalent rectangular shape (by introducing in-fill concrete blocks) eliminates the debonding due to the concave shape. The efficiency of the U-shaped CFRP shear reinforcement increases further by providing anchorages in the compression zone. Based on the given observations, U-shaped CFRP configurations anchored in the compression zone could be a more reliable solution. As far as tested in this work, the spike anchor appeared most promising.

Note that these recommendations should be given careful consideration, as these findings have not yet been extensively validated by research.

## 5. Conclusions

The current experimental study focussed on comparing two strategies for shear strengthening of I-shaped girders revealed the following important aspects.

1. FRP shear strengthening configurations along the shape of the I-girders are susceptible to early debonding at the web-flange corners resulting in only marginal or no increase in the shear capacity. In order to increase their efficiency, proper anchorage systems are required;
2. The devised specimen configuration as well as the test setup proved to be effective for the comparative study of different shear strengthening strategies and configurations for I-shaped girders;
3. U-shaped configurations with in-fill blocks (of both types) eliminated the debonding due to the concave shape of the I-section and exhibited higher ultimate loads compared to the equivalent I-shaped configurations;
4. Unanchored configurations with in-fill aerated concrete blocks as well as concrete in-fill blocks exhibit similar ultimate loads, though with differences in the failure mode. For the anchored U-shaped configurations, the concrete in-fill blocks outperformed those with aerated concrete;
5. The CFRP spike anchors in the I-shaped configurations transformed CFRP debonding to CFRP rupture/anchorage failure and increased the efficiency up to 36% for the tested configurations. Similar anchorages in the U-shaped configurations (along with the in-fill blocks) increased the efficiency by 43–54%;
6. Compared to CFRP spike anchors, the bolted anchors appeared less effective for the tested U-shape configurations, with an increase of ultimate load of 22–44% instead of 43–54%;
7. Among all the anchored configurations, the resistance against crack opening was the highest for the I-shaped CFRP configuration, whereas, with respect to the ultimate load the U-shaped configuration with concrete in-fill blocks was the most effective.

## Figures and Tables

**Table 1:** Test matrix (round #1: stirrup + 1 CFRP layer; round #2: no stirrup + 2 CFRP layers).

Group	Specimen Designation	Strengthening Configuration	In-Fill Block	Anchor Type
G1	I-NA	I	No	No
	I-SA	I	No	CFRP spike
G2	U-ACB-NA	U	ACB	No
	U-ACB-MA	U	ACB	Bolted plate
	U-ACB-SA	U	ACB	CFRP spike
G3	U-CB-NA	U	CB	No
	U-CB-MA	U	CB	Bolted plate
	U-CB-SA	U	CB	CFRP spike

**Table 2:** Strength of concrete.

Concrete for	Compressive Strength (MPa)	Flexural Tensile Strength (MPa)	Splitting Tensile Strength (MPa)
I-sections	63.0	6.4	4.6
Concrete blocks	63.0	5.6	5.0
Aerated concrete blocks	4.0	-	-

**Table 3:** Mechanical properties of steel stirrups and CFRP strips.

Reinforcement Type	Nominal Size (mm)	Yield Strength (MPa)	Tensile Strength (MPa)	Ultimate Strain (%)	Elastic Modulus (GPa)
Stirrups	Ø6.0	570	682	4.7	200
CFRP	150 × 0.113	-	4400	1.8	240

**Table 4:** Ultimate loads and failure modes.

Group	Specimen Designation	Ultimate Load (kN)	Strengthening Degree with Respect to ‘NA’ Specimen of That Group	Strengthening Degree with Respect to ‘I-NA’	Failure Mode
G1	I-NA	86.0	-	-	FRP debonding at the lower web-flange corner
	I-SA	116.6	1.36	1.36	FRP strip and anchor rupture at the lower web-flange corner
G2	U-ACB-NA	96.4	-	1.12	FRP debonding at the top flange
	U-ACB-MA	105.0	1.09	1.22	FRP debonding with slippage under the bolted steel plate anchor
	U-ACB-SA	123.3	1.28	1.43	FRP debonding and rupture of top flange due to pullout of spike anchors
G3	U-CB-NA	95.5	-	1.11	FRP debonding started over the crack and extended over the full height
	U-CB-MA	123.7	1.30	1.44	FRP debonding with slippage under the bolted steel plate anchor
	U-CB-SA	131.3	1.37	1.53	FRP rupture under the spike anchors

## Data Availability

The original contributions presented in the study are included in the article, further inquiries can be directed to the corresponding author.
